# The effect of combining action observation in virtual reality with kinesthetic motor imagery on cortical activity

**DOI:** 10.3389/fnins.2023.1201865

**Published:** 2023-06-13

**Authors:** Kishor Lakshminarayanan, Rakshit Shah, Sohail R. Daulat, Viashen Moodley, Yifei Yao, Deepa Madathil

**Affiliations:** ^1^Neuro-Rehabilitation Lab, School of Electronics Engineering, Vellore Institute of Technology, Vellore, Tamil Nadu, India; ^2^Department of Chemical and Biomedical Engineering, Cleveland State University, Cleveland, OH, United States; ^3^Department of Physiology, University of Arizona College of Medicine – Tucson, Tucson, AZ, United States; ^4^Arizona Center for Hand to Shoulder Surgery, Phoenix, AZ, United States; ^5^Soft Tissue Biomechanics Laboratory, School of Biomedical Engineering, Med-X Research Institute, Shanghai Jiao Tong University, Shanghai, China; ^6^Jindal Institute of Behavioural Sciences, O.P. Jindal Global University, Sonipat, Haryana, India

**Keywords:** virtual reality, kinesthetic motor imagery, brain computer interface, electroencephalography, event-related desynchronization, machine learning

## Abstract

**Introduction:**

In the past, various techniques have been used to improve motor imagery (MI), such as immersive virtual-reality (VR) and kinesthetic rehearsal. While electroencephalography (EEG) has been used to study the differences in brain activity between VR-based action observation and kinesthetic motor imagery (KMI), there has been no investigation into their combined effect. Prior research has demonstrated that VR-based action observation can enhance MI by providing both visual information and embodiment, which is the perception of oneself as part of the observed entity. Additionally, KMI has been found to produce similar brain activity to physically performing a task. Therefore, we hypothesized that utilizing VR to offer an immersive visual scenario for action observation while participants performed kinesthetic motor imagery would significantly improve cortical activity related to MI.

**Methods:**

In this study, 15 participants (9 male, 6 female) performed kinesthetic motor imagery of three hand tasks (drinking, wrist flexion-extension, and grabbing) both with and without VR-based action observation.

**Results:**

Our results indicate that combining VR-based action observation with KMI enhances brain rhythmic patterns and provides better task differentiation compared to KMI without action observation.

**Discussion:**

These findings suggest that using VR-based action observation alongside kinesthetic motor imagery can improve motor imagery performance.

## 1. Introduction

Rehabilitation paradigms for patients seeking to improve their motor functions are increasingly employing brain computer interface (BCI) assistive devices. These devices utilize motor imagery (MI) training, the mental “rehearsal” of movement without the concomitant motor execution, since imagining a motor task elicits a short-lasting attenuation of rhythms within the alpha (8–13 Hz) and beta (13–25 Hz) frequency bands called event-related desynchronization (ERD) (Pfurtscheller and Neuper, 1997; Jeon et al., 2011; Seo et al., 2019). Such ERD is similar to the cortical activity during actual movement execution (Seo et al., 2019), making MI suitable for BCI-assisted rehabilitation for patients with motor deficits (Jackson et al., 2001). One approach to elicit MI is action observation, or the observation of movement of the task-related body part (Vogt et al., 2013; Gonzalez-Rosa et al., 2015; Ono et al., 2018). Observation of actions elicits robust activation of the mirror neuron network (MNN), as assessed by functional imaging studies (Rizzolatti, 2005). Furthermore, MNN activation has been shown to induce cortical plasticity as demonstrated by fMRI, PET, and TMS analyses (Jackson et al., 2003; Cramer et al., 2011; Nojima et al., 2012). These studies suggest that MI training through action observation may induce cortical plasticity via MNN activation.

A common method employed to elicit MNN activation is mirror therapy, where subjects observe the reflection of the actions made by one side of their body that provides an illusion of movement of the contralateral side that may have limited motor function (Deconinck et al., 2015; Herrador Colmenero et al., 2018). Studies have shown that MNN activation is enhanced by visual aid (Sakamoto et al., 2009; Eaves et al., 2014; Zhang et al., 2018), specifically, a study by Nagai and Tanaka (2019) showed participants having stronger ERD while observing their own hands performing a motor task as compared to observing another person’s hand or no movement while performing motor imagery. Such studies suggest a stronger ERD during MI in the presence of immersive visualization and a stronger ownership of the presented body (Alimardani et al., 2016; Penaloza et al., 2018; Škola and Liarokapis, 2018; Juliano et al., 2020).

Virtual reality (VR) headsets provide an immersive environment and have the potential to amplify body ownership (Slater, 2017; Tham et al., 2018; Choi et al., 2020). VR headsets have the ability to blur the lines between the real and virtual world, creating an environment that is perceived as real by the subjects. With these advantages, VR headsets have been frequently employed for action observation during motor imagery practice (Choi et al., 2020). VR has assisted MI in a VR-based neurofeedback system designed to help patients improve their MI performance for tasks involving arm or limb movements that used visualizations of graphical body movements that respond to the participant’s brain activity, providing feedback for the motor imagery process (Vourvopoulos and Bermúdez i Badia, 2016). Both MI and VR therapies share a common emphasis on cognitive movement, where MI requires sensory and perceptual processes (Lequerica et al., 2002), while VR engages participants in cognitive and motor activities simultaneously (Messier et al., 2007; Cole et al., 2012). Studies involving VR-based MI have largely targeted stroke survivors (Sirigu et al., 1996; Kimberley et al., 2006), where VR integrated into motor imagery-based rehabilitation protocols resulted in shorter rehabilitation time and improved recovery after injury in patients with stroke (Cameirao et al., 2012; Turolla et al., 2013). Faster rehabilitation time was also demonstrated in war veterans who participated in rehabilitation using the immersive VR-based Computer Assisted Rehabilitation Environment (CAREN) system (Isaacson et al., 2013). VR-based action observation has been shown to elicit improved cortical rhythmic patterns and spatially discriminating features compared to conventional action observation via computer monitors (Slater, 2017). Such data suggest that action observation in an immersive virtual reality environment may offer better cortical neuroplasticity compared to conventional action observation.

Research has shown that kinesthetic motor imagery, or the ability to imagine movement by means of proprioception, elicits an ERD response that correlates with actual execution of the motor task (Toriyama et al., 2018). Studies have shown that when MI is accompanied by an impression of muscle contraction and limb movement, there is an overlap of the task-specific cortical networks between imagination and execution of the task in question, implying that kinesthetic MI recruits similar neuronal circuits as movement itself (Stinear et al., 2006; Toriyama et al., 2018). According to Frith and Dolan (1997), KMI depicts an internal simulation of the anticipated sensory effects of real task execution in the absence of sensory input. The somatotopic structure of the cerebellum and the neural connections that connect it to the sensorimotor cortex support the idea that the cerebellum plays a part in the development of forward models of anticipated sensory feedback during real and imagined movement (Miall and Wolpert, 1996). However, the majority of studies have performed kinesthetic MI through auditory or abstract visual cues. Very little information is available on the role of presence of action observation in virtual reality during kinesthetic motor imagery in enhancing ERD response. The strong body ownership resulting when subjects view the motor task inside an immersive VR environment has the potential to enhance the kinesthetic imagination of the body parts involved in performing the motor task in question.

Although simple MI-based training is frequently used in MI-based BCI training, such as the left and right hand or foot, it is mostly ineffective in patients with impaired motor function, such as those who have had a paralyzing stroke on one side of the body (Benzy et al., 2020; Dahms et al., 2020). These patients merely need to enhance their motor skills in the afflicted limb. Simple MI is constrained in these situations since it cannot distinguish between the components of the same limb. However, complex MI using compound imaging utilizing a single limb has been created to replace traditional simple MI (Ma et al., 2019) since it is more crucial to use the neurofeedback from the affected limb than of both limbs in rehabilitation (Benzy et al., 2020; Dahms et al., 2020). When it comes to the targeted training of a single afflicted limb, complex MI offers an edge over traditional simple MI. Additionally, the compound MI from a single limb has increased the number of instructions that may be used during MI-based BCI (Yi et al., 2016), which would enable the development of more advanced and functional prostheses or assistive devices.

Therefore, the purpose of this study was to determine the effect of combining VR-based action observation with kinesthetic motor imagery of hand movements on the cortical activity and task classification performance for a complex MI. To achieve this, we examined brain activities using electroencephalography (EEG) during kinesthetic MI with versus without action observation of the imagined tasks in a VR environment. The complex MI employed here was imagining three different hand tasks using a single given limb. Furthermore, machine learning techniques were applied to discriminate elicited responses from the sensorimotor cortex during different MI tasks. It was hypothesized that VR-based kinesthetic MI would elicit a greater neuronal response and spatially discriminating cortical rhythmic patterns compared to non-visual aided kinesthetic MI.

## 2. Methods

### 2.1. Subjects

Fifteen healthy right-handed adults (six females and nine males) with a mean age of 28 ± 4 years participated in the study. The handedness of each subject was assessed using a verbal self-report assessment where we questioned each subject the hand the subject use for common activities such as writing, throwing, dealing cards, and using an eraser (Coren, 1989). The number of subjects was chosen based on previous similar studies with MI (Decety and Jeannerod, 1995; Chiarelli et al., 2018). A post-hoc power analysis yielded a power of 0.8 (paired *t*-test power). All participants verbally disclosed that they had no history of upper limb injury or musculoskeletal or neurologic disorders. All participants had no prior experience with motor imagery or VR. The study protocol was approved by the Vellore Institute of Technology Ethical Committee for Studies on Human Subjects (VIT/IECH/IX/Mar03/2020/016B). Participants read and signed a written informed consent form approved by the Institutional Review Board before participating in the experiment.

### 2.2. Equipment

Allengers’ Virgo (Allengers Medical Systems, Chandigarh, India) EEG system was used to record EEG signals by placing an EEG cap on the scalp of each participant. EEG signals were recorded from 20 electrodes (FP1, FPz, FP2, F7, F3, Fz, F4, F8, T3, C3, Cz, C4, T4, T5, P3, Pz, P4, T6, O1, O2) placed according to the international 10–20 system. Ground and reference electrodes were located at FPz and Fz, respectively. Each electrode site was hydrated using a conductive gel to keep the impedance under 5 kΩ and obtain high-quality data. EEG data was recorded continuously at 250 Hz during the experiment.

3D avatars that resemble each participant were modeled in Blender software (Blender Foundation, Amsterdam, Netherlands) by 3D scanning the face and taking body measurements for each participant. Each avatar was then animated in Blender to perform three different right-hand tasks, namely drinking from a cup, flexion-extension of the right-hand wrist, and grabbing a cup. The avatars were then exported to a 3D virtual environment and gamified to perform the animation multiple times using Unity game engine (Unity Technologies, San Francisco, CA, USA). Participants wore an Oculus Rift-S (Oculus VR, Menlo Park, CA, USA) VR headset after wearing the EEG cap so that the VR headset was on top of the EEG cap. The VR headset displayed the graphical scenario with the avatar performing the hand tasks in an immersive VR environment to the participants.

### 2.3. Experimental design

**Figure 1:**
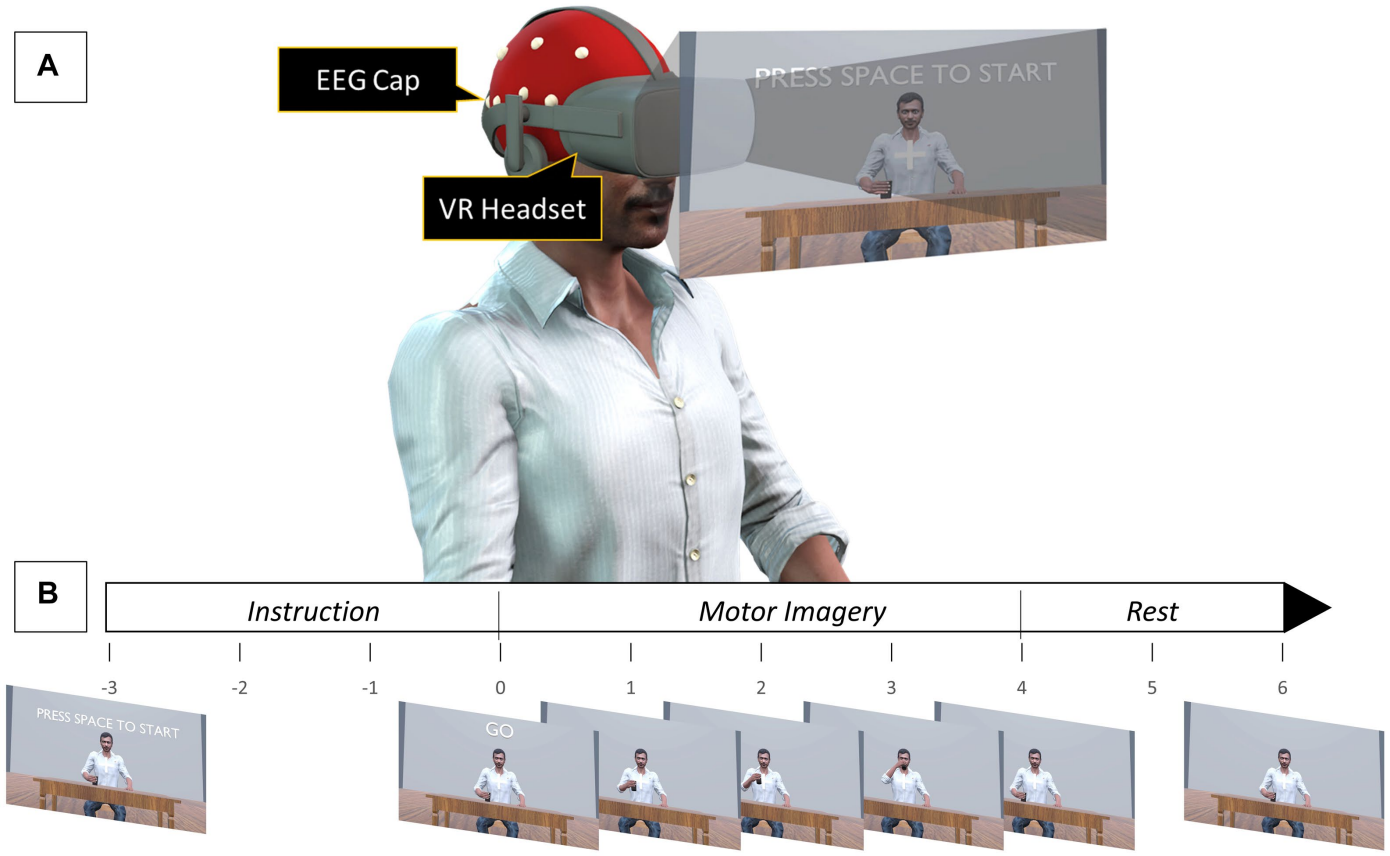
**(A)** Experimental setup for VR-KMI. For NVA-KMI, the VR headset was removed, and subjects were asked to close their eyes. **(B)** Timeline of a single motor imagery task.

To evaluate whether using a VR environment to provide action observation during kinesthetic motor imagery would enhance MI-induced cortical activity two experiments were performed, namely VR-based kinesthetic motor imagery (VR-KMI) and Non-visual aided kinesthetic motor imagery (NVA-KMI). The experimental design was adapted from a previous study by Choi et al. (2020), where EEG was recorded while subjects performed action observation with the visual scenario presented through either a monitor or a VR headset. The experiments were conducted in a quiet room with minimal environmental distractions. Participants were instructed to sit comfortably in a chair with their arms on the arm rest (Figure 1A). Subjects were given clear instructions to not make any movements during the experiment, including any movement related to the tasks being imagined and also eye blinks and head movement to avoid artifacts in the EEG. Each MI experiment consisted of three blocks with one block each for the three different right-hand tasks (Drinking, Flexion-Extension, and Grabbing). Each block had five sessions with ten consecutive trials of MI in each session. Adequate rest was provided between each session. Each MI trial consisted of a 3-s instruction period followed by a 4-s MI period then a 2-s rest. Overall, each participant performed 150 MI trials for each experiment, with 50 trials per hand task. The order of the two experiments and the order of the blocks within each experiment were randomized for each participant. EEG was recorded continuously during an entire block.

#### 2.3.1. Experiment 1 – VR-KMI

In VR-KMI, each participant performed kinesthetic MI while they watched a 3D avatar that resembled the participant through a VR headset, perform the three right hand tasks. Participants were asked to observe their 3D avatar performing the task and imagine kinesthetically the same movement. Instructions were given for each of the three tasks to ensure proper performance of KMI. For instance, the instruction given for the flexion-extension task was “imagine flexing and extending your wrist in time with the animation, and the feeling that this produces.” A practice run with 10 trials was performed to make the participants comfortable with imagining the task at the pace it was performed by the avatar. The practice session included actual execution of the tasks for the first 5 trials and then imagining it for the remaining trials. At the beginning of each session, a countdown of 3 s was displayed before the first MI trial started (Figure 1B). An auditory cue was also given to indicate the start of the trial.

#### 2.3.2. Experiment 2 – NVA-KMI

In this experiment, participants were asked to imagine kinesthetically the same three right hand tasks without any visual aid. Prior to NVA-KMI, participants were shown an animation video of their 3D avatar performing the three hand tasks to aid them understand how each task was performed and the pace at which it was performed. Subjects were also made to perform a practice run of 10 trials before the actual data collection started, where they executed the task movement for the first five trials and then practiced imagining the trials for the remaining five trials. During the actual experiment, participants were asked to close their eyes and were given an auditory cue to indicate the start of each trial. Participants were instructed to form an impression of their own right hand performing the task after they heard the auditory cue. The instruction given, for instance the grabbing task was “imagine grabbing the cup and letting it go at the pace you practiced, and the feeling that this produces.”

### 2.4. EEG analysis

#### 2.4.1. Pre-processing

The EEG data analysis was performed using the toolboxes in MATLAB (The MathWorks, Natick, MA). ERD analysis was performed using the EEGLAB toolbox, while the task discrimination analysis was performed using the Neural Network toolbox. The recorded EEG data was pre-processed by band pass filtering between 1 and 50 Hz and re-referencing the data by applying the average reference over all the electrodes. Independent component analysis (ICA) was performed on the data to remove sources of artifacts using the ADJUST algorithm (Mognon et al., 2011). The data was then epoched from −1000 to 6000 ms relative to the start for each MI trial. This pre-processed EEG data was used for further analyses.

#### 2.4.2. Time-frequency analysis

The C3 electrode was used for the ERD analysis corresponding to the three different MI hand tasks since the left sensorimotor area corresponding to the C3 electrode correlates with the contralateral right-hand activities. Time-frequency analysis was performed to find the Event-related spectral perturbations (ERSP) for the EEG data from C3 electrode. The ERSP provides the dynamic change in the amplitude of the linearly spaced frequency spectrum between 1 and 50 Hz as a function of the time length of the epoch. The time length of the epoch was linearly spaced to 200 time points. The ERSP provided the neural activity during a MI task in the form of an ERD, or a decrease in the frequency amplitude in the time period the task was imagined.

The ERSP for individual epochs were determined and normalized by dividing by their respective baseline spectra. Average ERSP was finally calculated by averaging all the normalized ERSP for each participant. The alpha band (8–12 Hz) and beta band (13–30 Hz) were chosen for the ERD analysis since previous studies have shown ERD activity being largely elicited in these bands during MI (Pfurtscheller and Neuper, 1997; Jeon et al., 2011; Lakshminarayanan et al., 2023). The alpha and beta band ERD activity was calculated by averaging over each frequency band. Specifically, the ERD were ERSP averaged over the 4-s MI period (0–4000 ms) for each of the two frequency bands. The ERD results of the C3 electrode were calculated for each of the three tasks in both VR-KMI and NVA-KMI conditions. For statistical analysis, repeated-measures ANOVAs were performed on the task-related average ERD activity in alpha and beta bands separately. The independent variables included experiment (VR-KMI vs. NVA-KMI), and task (Drinking, Flexion-Extension, and Grabbing). The statistical analysis was performed using SigmaStat 4.0 (Systat Software Inc., San Jose, CA, USA). An *α* level of 0.05 was considered for statistical significance.

#### 2.4.3. Discriminant analysis

Neural activity discrimination of the three tasks during the two experiments was evaluated by constructing machine learning models. For this purpose, the MATLAB nprtool module of the Neural Network Toolbox was utilized. The toolbox uses a two-layer feedforward network, with a scaled conjugate gradient backpropagation algorithm-based learning process (Beale et al., 2010). Discriminant characteristics that are associated with each task were extracted from the ERSP data for training and testing the classifier. A 3-s [1000–4000 ms] window of ERSP data was extracted for each trial of each task. The extracted window corresponded to the time period ERD occurred during MI of the tasks. The extracted ERSP data was averaged across two frequency bins – alpha (8–12 Hz) and beta (13–30 Hz) to obtain alpha and beta ERD activity, respectively. The extracted features were then concatenated. The input data to the neural network consisted of average alpha and beta ERD activity over time with 150 data points each corresponding to the ERD for each of the 50 trials from the three tasks. A target vector with a dimension of 3 × 150 representing the three tasks’ labels (Drinking, Flexion-Extension, and Grabbing) was also inputted to the neural network. The input layer consisted of 150 neurons (N) corresponding to the 150 data points, while the output layer had 3 neurons (M) for the three task classes. The number of neurons in the hidden layer was set at 22 using the following formula (Fadiyah and Djamal, 2019).


$$\sqrt{N\;x\;M}$$

The neural network was trained on 70% of the data and was validated and tested on the remaining 30% of the data with 15% each for the validation and testing. Once the hidden layer neurons were determined, the neural network was run 100 times to reduce the influence of the randomly chosen training data. The accuracy percentages from the 100 runs were averaged to obtain the final classification accuracy percentage. To conduct the statistical analysis, a paired *t*-test was performed on the classification accuracy from the two experiments. Statistical analysis including power analysis was performed in SigmaStat 4.0 (Systat Software Inc., San Jose, CA, USA).

## 3. Results

**Figure 2:**
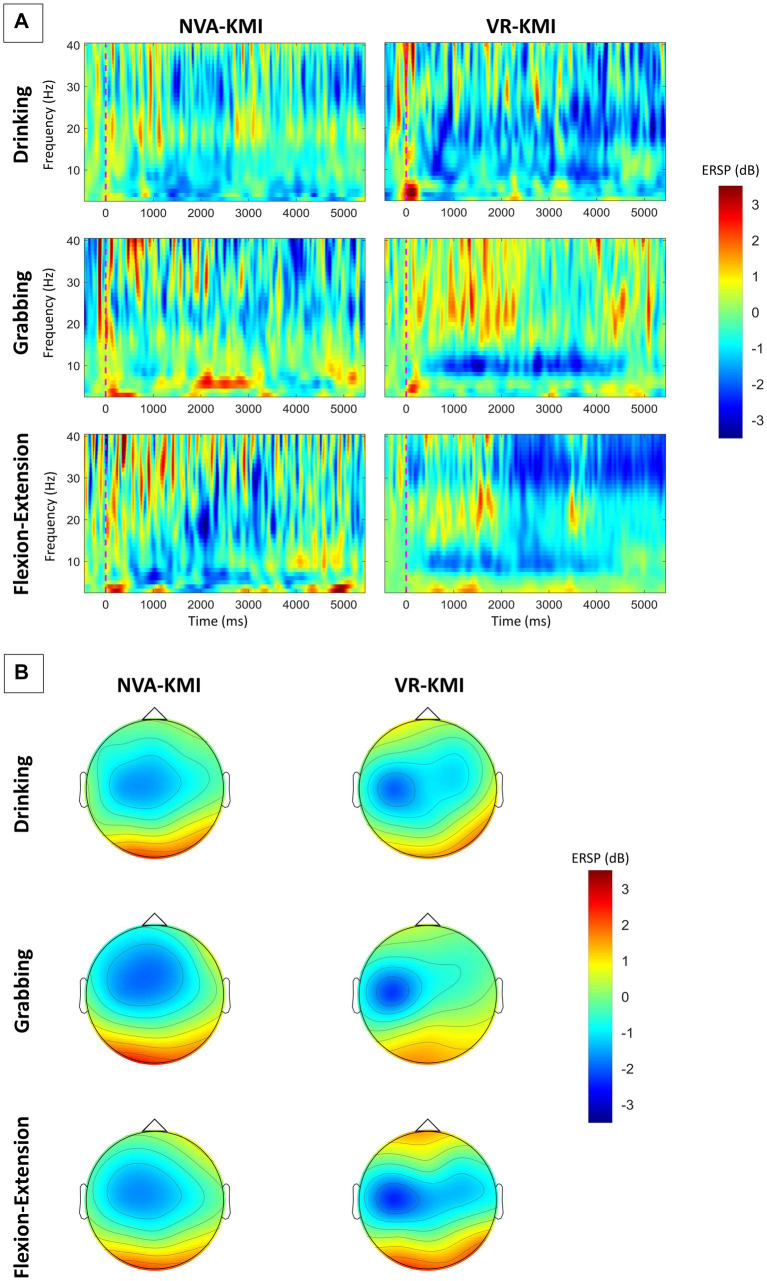
**(A)** Averaged time-frequency maps of all participants at C3 electrode. **(B)** Averaged topographical distribution of power during MI for alpha and beta bands combined (8–30 Hz). Blue indicates ERD.

In the current study, subjects performed trials with periods of kinesthetic MI while either keeping their eyes closed (NVA-KMI) or observing action in VR (VR-KMI). Figure 2 shows the average time-frequency maps of all subjects at the C3 channel for all three hand tasks under both experiment conditions (VR-KMI and NVA-KMI). The time-frequency maps (Figure 2A) clearly show a long-lasting event-related desynchronization (ERD) during the task performance (0–4000 ms) with larger ERD amplitude in VR-KMI condition compared to NVA-KMI. Furthermore, to study the activation of the sensorimotor area, the spatial patterns over the 4-s motor imagery period during VR-KMI and NVA-KMI for the three hand tasks in the alpha and beta bands combined (8–30 Hz) are shown in Figure 2B. The mean EEG potential was topographically located in the contralateral sensorimotor area consistent with previous studies (Simonyan et al., 2012). VR-KMI showed a stronger potential at the contralateral sensorimotor area compared to NVA-KMI at both frequency bands for all three tasks.

Statistical analysis was performed to test if the differences between the two experiment conditions were significant. A repeated-measures ANOVA was applied to study the differences between VR-KMI vs. NVA-KMI. As a first step, the assumptions made by the ANOVA on the ERD results were verified. There were no significant outliers that were identified. Shapiro–Wilk normality test indicated that the normality was not violated for all groups (*p* > 0.05) and the results followed a normal distribution. Sphericity is the condition where the variances of the differences between experiment conditions are equal. A Brown-Forsythe test was performed to test this assumption and the results confirmed equal variance (*p* > 0.05).

**Figure 3:**
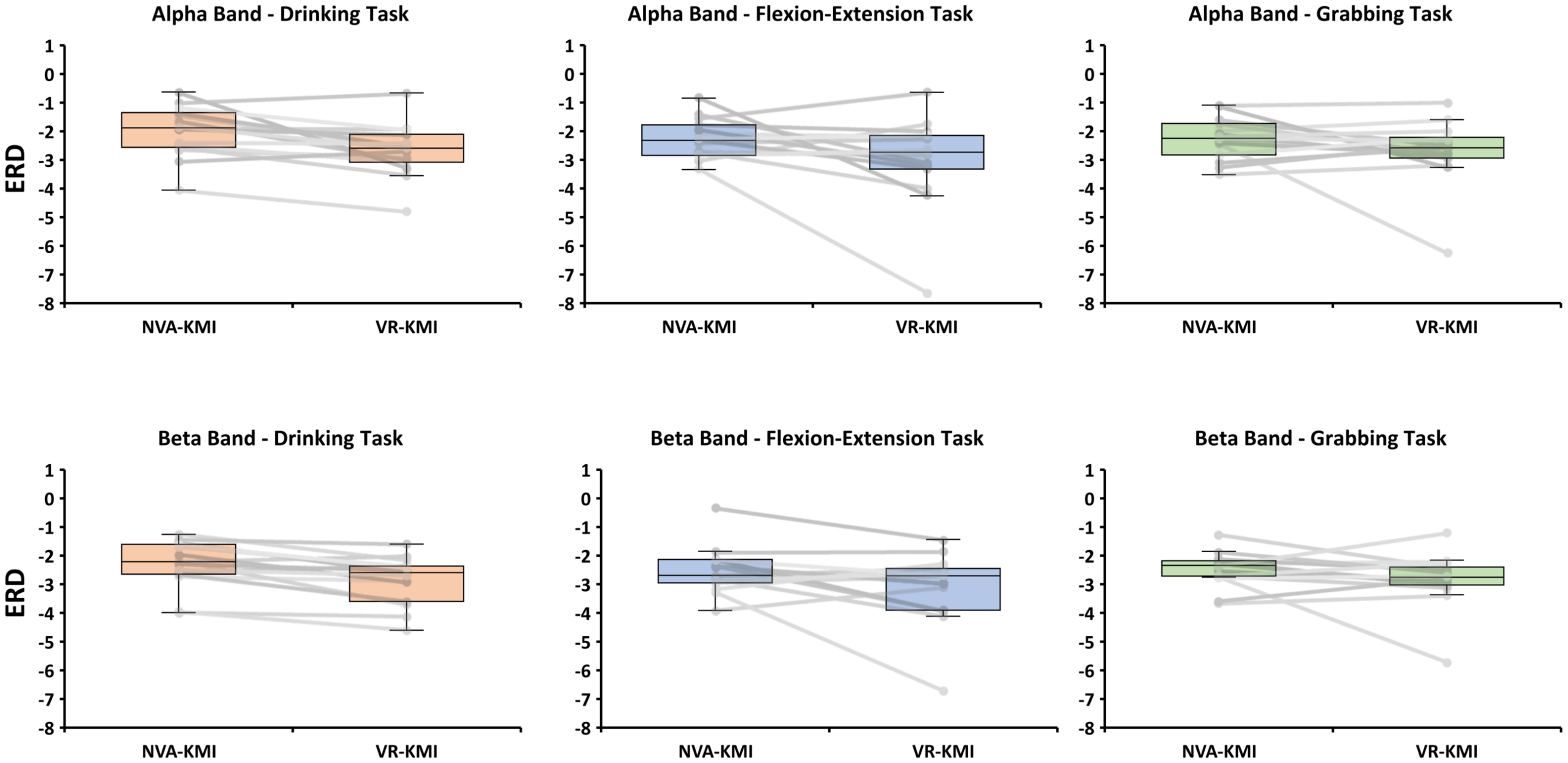
Boxplot and individual data points of ERD for all participants from alpha and beta bands for both experiment conditions (NVA-KMI and VR-KMI) for each task.

ERD during motor imagery was compared between two experiment conditions namely VR-based kinesthetic motor imagery (VR-KMI) and Non-visual aided kinesthetic motor imagery (NVA-KMI) (Figure 3). In the alpha band, repeated measures ANOVA showed that ERD significantly differed by experiment (VR-KMI vs. NVA-KMI, *p* = 0.034) but not by task (Drinking, Flexion-Extension, and Grabbing, *p* = 0.286). Furthermore, the interactions were not found to be significant (*p* = 0.658). Specifically, the ERD (dB) increased in VR-KMI compared to NVA-KMI (*M* = −2.8, SD = 1.2 for VR-KMI, *M* = −2.2, SD = 0.75 for NVA-KMI). The powers of performed tests with alpha = 0.05 was 0.5 for the experiment and 0.09 for the task.

In the beta band, the repeated measures ANOVA showed similar results as the alpha band with ERD significantly differing between the experiments (*p* = 0.016) but not by activity (*p* = 0.509). The ERD (dB) showed a significant increase (decrease in EEG power) in VR-KMI (*M* = −2.9, SD = 1.0) compared to NVA-KMI (*M* = −2.4, SD = 0.7) (Figure 3B). ERD did not differ by task or the interaction between task and experiment (*p* = 0.741). The powers of performed tests with alpha = 0.05 was 0.7 for the experiment and 0.05 for the task.

The classification accuracy was defined as the percentage of correctly classified tasks for each experiment condition. Table 1 shows the classification percentage for the two experiments for each participant. One-sample t-tests were performed on the accuracy values from each of the two conditions (VR-KMI and NVA-KMI) and the sample mean of the group exceeded the hypothesized mean by an amount that is greater than would be expected by chance for both conditions (*p* < 0.05). A Shapiro–Wilk normality test indicated that the values were normally distributed (*p* > 0.05). A paired *t*-test revealed that VR-KMI showed a significantly higher (*p* = 0.001) accuracy percentage (*M* = 61.9%, SD = 8.3%) compared to NVA-KMI (*M* = 54.3%, SD = 6.0%) indicating that ERD during VR based kinesthetic motor imagery showed a higher discrimination between the three tasks namely Drinking, Flexion-Extension, and Grabbing compared to kinesthetically imagining the tasks with no visual cue.

**Table 1:** Classification accuracy percentage for each subject.

Subject	VR-KMI	NVA-KMI
1	80.3%	52.9%
2	52.9%	62.6%
3	47.9%	43.5%
4	57.8%	51.3%
5	56.6%	50.9%
6	68.1%	53.6%
7	62.4%	57.2%
8	77.6%	69.8%
9	62.2%	51.1%
10	60.8%	50.1%
11	59.4%	55.8%
12	60.8%	55.8%
13	58.5%	56.5%
14	62.2%	51.3%
15	61.8%	52.1%

## 4. Discussion

In the current study, repeated kinesthetic motor imagery was performed with and without action observation in an immersive, graphical virtual reality scenario to investigate the effect of combining VR-based action observation with kinesthetic rehearsal of hand tasks on motor imagery performance in healthy adults. The ERD in the alpha and beta bands and the task discrimination analysis using neural networks from the current study have provided evidence that perceiving actions through VR vs. performing kinesthetic motor imagery with eyes closed produce different motor imagery-related cortical activities. Specifically, we observed that participants showed a larger kinesthetic MI-induced ERD response with VR compared to without any visual presentation. The results from the study not only confirmed that repeated MI training kinesthetically have an effect on the cortical activity as seen in other studies (Stinear et al., 2006; Toriyama et al., 2018), and VR-based action observation is more effective in improving motor imagery classification performance as suggested by other studies (Simonyan et al., 2012; Maselli and Slater, 2013; Sun et al., 2016; Waltemate et al., 2018), but also confirmed that combining the immersive action observation in VR and kinesthetically performing motor imagery resulted in eliciting a greater ERD response and an increased spatial discrimination of hand task-related brain activity compared to only kinesthetic motor imagery without any visual presentation.

The ERD during motor imagery was investigated for VR-KMI and NVA-KMI for right-handed tasks from the contralateral left motor cortex (C3). Figure 2 illustrates that ERD activity was primarily located contralaterally across all tasks and experimental conditions. According to previous research, ERD activity especially in the beta band is associated with motor tasks (Khanna and Carmena, 2015). The ERD activity increased significantly during the kinesthetic motor imagery period in both alpha and beta bands. The significant difference in the ERD response between VR-KMI and NVA-KMI only during motor imagery indicated that the increased ERD during VR-KMI was elicited by the better motor imagery performance. Furthermore, the ERD response was not significantly different across the three tasks (Drinking, Flexion-Extension, and Grabbing) for both VR-KMI and NVA-KMI indicating that MI performance was not task dependent. The results from the current study suggest that including VR-based action observation during kinesthetic motor imagery enhanced performance of the imagery.

The current study focused on whether the inclusion of action observation with kinesthetic motor imagery would be effective. The ERD and the task discrimination results have verified our hypothesis by showing an increased ERD and higher task classification accuracy. Specifically, the higher classification accuracy between the three hand tasks during VR-KMI from the same single electrode (C3) has implications in rehabilitation and especially brain-computer interface applications. The major component that contributed to the enhancement in motor performance is action observation in the VR environment, which has been reported in previous studies to improve illusion and embodiment (Stecklow et al., 2010; Liang et al., 2016; Roth et al., 2017; Choi et al., 2020). Although, repeating motor imagery kinesthetically in both VR-KMI and NVA-KMI conditions elicited ERD response and showed task discriminability, combining kinesthetic MI with action observation in VR showed a better MI performance. Incorporating both action observation (AO) and MI, the experimental design of the present study aimed to elicit a greater desynchronization, as previously demonstrated by Berends et al. (2013). In order to ensure participants engaged in both AO and MI simultaneously, explicit instructions were provided to encourage kinesthetic imagining of observed movements. Vogt et al. (2013) have suggested that AO and MI training should be used in conjunction, rather than viewed as separate treatment methods. This recommendation was based on a review of several studies. Thus, congruent VR-based action observation and kinesthetic motor imagery may improve motor imagery performance at a less time cost compared to no visual presentation during kinesthetic motor imagery practice.

The study has some limitations. Although much research has confirmed that using an immersive graphical scenario affects motor imagery performance (Liang et al., 2016; Roth et al., 2017), studies have also shown that different visual scenarios may result in different MI-induced cortical response (Lebon et al., 2012; Choi et al., 2020). Our study used a graphical scenario that differed from other studies, where the subjects saw a 3D avatar of themselves performing the motor tasks akin to a third person perspective. Therefore, the findings from our study could not be generalized to any graphical scenario presented via VR. Secondly, the study has a relatively small sample size. Although the trials were repeated, there still were variations in each participant’s performance. Furthermore, the study lacked an objective method to measure any erroneous movement the subjects might have made during the imagery tasks. Although the subjects were given instructions to not make any movement during the experiment, we lacked EMG to measure any muscle activation during MI. Thus, the results from the current study need to be interpreted carefully. Lastly, the study was limited in the amount of memory required for running the machine learning algorithm, restricting the neural network to just one electrode and two features (alpha and beta ERD). The limitation led to our classification percentage though significantly different between the two experimental conditions, still being relatively low compared to other studies. Future studies will focus on recruiting more features in the neural network.

## 5. Conclusion

Our study focused on the combined effect of action observation and kinesthetic motor imagery, unlike previous research, which has largely focused on comparing visual scenarios for action observation during motor imagery or kinesthetic motor imagery versus action observation. With other studies showing the positive effects of immersive and enhanced illusion through VR on improving motor imagery performance, we investigated whether observing actions of a 3D avatar that resembled each participant in VR could improve the already effective kinesthetic motor imagery.

We have examined two different aspects of brain activity during kinesthetic motor imagery: the oscillatory rhythm changes in the motor imagery-related brain regions, and the discrimination between tasks based on the spatial characteristics of the brain signals, explored using a machine learning algorithm. The results from the ERD and discrimination analyses showed that VR-based kinesthetic motor imagery resulted in higher oscillatory changes and greater spatial discrimination in the neural activity. Thus, the current study proposes using VR for action observation along with kinesthetic motor imagery for an enhanced motor imagery performance.

## Data availability statement

The original contributions presented in the study are included in the article/supplementary material, further inquiries can be directed to the corresponding author.

## Ethics statement

The study protocol was approved by the Vellore Institute of Technology Review Board (VIT/IECH/IX/Mar03/2020/016B). Subjects read and signed a written informed consent form approved by the Institutional Review Board before participating in the experiment.

## Author contributions

KL and RS analyzed the data and wrote the manuscript with the other authors contributing to revisions. KL recruited the subjects and conducted preliminary tests for eligibility, collected data from the subjects, and worked as the guarantor and, as such, had full access to all the data in the study and takes responsibility for the integrity of the data and the accuracy of the data analysis. KL, RS, SD, VM, YY, and DM designed the study. All authors contributed to the article and approved the submitted version.

## Funding

This research was supported by the Department of Science and Technology, India (Grant number SRG/2021/000283).

## Conflict of interest

The authors declare that the research was conducted in the absence of any commercial or financial relationships that could be construed as a potential conflict of interest.

## Publisher’s note

All claims expressed in this article are solely those of the authors and do not necessarily represent those of their affiliated organizations, or those of the publisher, the editors and the reviewers. Any product that may be evaluated in this article, or claim that may be made by its manufacturer, is not guaranteed or endorsed by the publisher.

## Abbreviations

MI: Motor Imagery
KMI: Kinesthetic Motor Imagery
VR: Virtual Reality
NVA: Non-Visual Aided
EEG: Electroencephalography
ERD: Event-related Desynchronization
AO: Action Observation
